# Lignins and Their Derivatives with Beneficial Effects on Human Health

**DOI:** 10.3390/ijms18061219

**Published:** 2017-06-07

**Authors:** Maria Pilar Vinardell, Montserrat Mitjans

**Affiliations:** Department of Biochemistry and Physiology, Faculty of Pharmacy and Food Sciences, Universitat de Barcelona, Avinguda Joan XXIII 27-31, 08028 Barcelona, Spain; montsemitjans@ub.edu

**Keywords:** lignin, human health, applicability, antioxidant capacity, antiviral effect

## Abstract

A review of the pharmacological applications of lignins provides evidence of their protective role against the development of different diseases. In many cases, the effects of lignins could be explained by their antioxidant capacity. Here, we present a systematic review of the literature from the period 2010–2016 which provides information concerning new applications of lignins derived from recent research. The most promising findings are reported, including the methodologies employed and results obtained with lignins or their derivatives which may improve human health. We highlight potential applications in the treatment of obesity, diabetes, thrombosis, viral infections and cancer. Moreover, we report both that lignins can be used in the preparation of nanoparticles to deliver different drugs and also their use in photoprotection.

## 1. Introduction

Lignins are present in significant amounts in plants, accounting for 15–25% (*w/w*) of herbaceous biomass. They consequently represent a large amount of the waste generated by different industries that use plant matter.

Nowadays, most commercial lignin is obtained as a by-product from lignocellulose treatments performed during pulp and paper processing, in the form of lignosulfonates and kraft lignins. The most common use of these lignins is in energy production, with only a small fraction being used for other commercial applications. This is due, in part, to the dispersing, binding and emulsifying properties of lignins. However, considerable effort is being made to find new applications of lignins, in order to add extra value to them while at the same time potentially diversifying biorefinery practices [1]. Different strategies have thus been developed to valorize lignins, such as ultrafiltration as a fractionation process to separate lignin fractions of different molecular weights [2].

The heterogeneity of the lignin structure makes it difficult to isolate a specific product, and in many cases the result of fractionation is a mixture of products. Furthermore, due to the complexity of the lignin structure and the effects of pretreatments on it, identifying and extracting chemicals from lignin requires extensive characterization to understand its polymeric properties and linkages, as well as the properties of the functional groups connected to the aromatic ring. The components of the lignin macromolecule together with its characterization suggest the predominance of antioxidant monomers [3]. A recent study found that low-molecular-weight monomers derived from hydroxycinnamic acids and guaiacyl units are responsible for the antioxidant properties of lignins [4].

**Figure 1:**
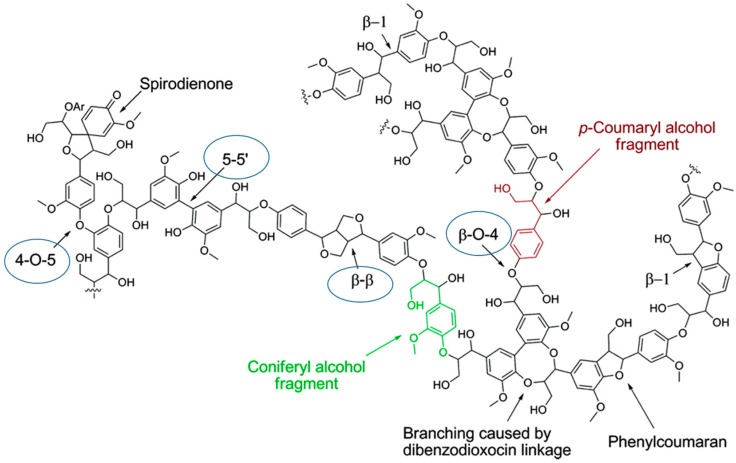
Schematic representation of a softwood lignin structure showing the different linkages. (Reprinted with permission from [5]).

The precise chemical structure of lignin is not known, both because of its complex polymeric nature and also due to the degree of random coupling involved in the arrangement of the macromolecule. Considerable work has been done on the detailed structural characterization of these complex natural polymers to gain an understanding of their structure and function. The composition, molecular weight, and amount of lignin differ from plant to plant, with lignin abundance generally decreasing in the order: softwoods > hardwoods > grasses. The components are joined by β-*O*-4, 5−5, β-5, 4-*O*-5, β-1, dibenzodioxocin, and β−β linkages, among others, of which the β-*O*-4 linkage is dominant, accounting for more than half of the linkage structures of lignin [5] as shown in Figure 1.

Lignin is a very good candidate for the development of new materials due to the presence of phenolic and aliphatic hydroxyl groups in its structure. These confer considerable potential for chemical modification [6] on the macromolecule.

Alkali lignin is currently the largest lignin class to have been produced. It a co-product of biofuel and the paper industry which is separated from fibers by chemical pulping (mainly soda and sulfite) but is of less value than the primary products [7].

Some old studies show that lignins are involved in different biological activities. These include reducing serum cholesterol by binding to bile acids in the intestine [8], and preventing tumor development as demonstrated in rats exposed to an intestinal carcinogen 3,2-dimethyl-4-ami-nobiphenyl and fed a lignin diet [9].

We previously reviewed the pharmacological applications of lignins and different products obtained from them; our results were published in 2012 and provide evidence of lignans playing a protective role against the development of different diseases [10]. In many cases, the effects of lignins could be explained by their antioxidant capacity.

Here, we now report a systematic review of the literature over the period 2010–2016 which provides information on new applications of lignins derived from recent research. We give details of the most promising findings, describing the methodologies employed and the results obtained with lignins or their derivatives which may improve human health.

## 2. Pharmacological Activities of Lignins and Their Derivatives

### 2.1. Lignins and Lignosulfonic Acid for the Treatment of Diabetes

Lignins can be obtained via different processes. One strategy is to obtain biomodified alkali lignin extracted from the deciduous plant *Acacia nilotica*. The extracted alkali lignin can then be subjected to microbial biotransformation by the ligninolytic fungi *Aspergillus flavus* and *Emericella nidulans*. The results obtained using this method demonstrate that the structure and functional modifications of the lignin significantly affect its antioxidant bioefficacy and reduce its antidiabetic activities [11]. However, the modified alkali lignin showed significant in vitro α-amylase inhibitory activity [12], thereby indicating that it potentially has anti-hyperglycemic properties. Moreover, the functional and structural modifications of alkali lignin altered the efficiency of its binding with the glucose molecule. This affected its movement across the cellular membrane and improved glycemic control by limiting postprandial glucose absorption. In conclusion, the antioxidant properties, α-amylase activity and in vitro glucose movement inhibition of *A. nilotica* lignin may offer a potential therapeutic source for the treatment of oxidative stress and diabetes [11].

*A. nilotica* is a deciduous tree in the Mimosaseae family which is predominantly found in central India. Different parts of *A. nilotica* have been studied in detail and are used as remedies for a variety of diseases in folk medicine. Among the traditional uses, we find treatment of various cancers of the mouth, bone and skin [13]. The health-promoting properties of this plant can be attributed to different biologically active compounds presents in it. Some researchers have studied the possibility of employing high-pressure-assisted solvent extraction and conventional solvent extraction methods to concentrate the antioxidant and anticancer constituents of *A. nilotica* wood and the alkali extract obtained from it. Eleven different lignin fractions, extracted from the wood of *A. nilotica* were studied. All the fractions displayed a significant capacity to scavenge nitric oxide, and hydroxyl and superoxide radicals. The extracted lignin fractions had high ferric ion reducing capacities and also possessed excellent antioxidant potential in a hydrophobic system (linoleic acid). Fractions extracted using a polar solvent had the highest iron (Fe^2+^) chelating activity, indicating their effect on the redox cycling of iron. Four lignin fractions showed higher cytotoxic potential towards the breast cancer cell line MCF-7, while being only slightly toxic to normal primary human hepatic stellate cells (HHSteCs). These findings suggest that lignin extracts from *A. nilotica* wood have a remarkable potential to prevent disease caused by the overproduction of radicals. They are also promising candidates as natural antioxidants and anti-cancer agents. These results suggest putative applications of lignin extracted from *A. nilotica* in the cosmetic, pharmaceutical and food processing industries, among others [14].

The effect of lignosulfonic acid on intestinal glucose absorption was also studied recently [15]. It was shown that lignosulfonic acid is a reversible and non-competitive inhibitor of α-glucosidase, indicating that it can bind to both the enzyme and enzyme-substrate complex. This inhibitory effect on α-glucosidase activity was enhanced by preincubation of the lignosulfonic acid with the enzyme, involving slow binding. The administration of glucose and lignosulfonic acid induced a delay in glucose uptake compared to administration of glucose alone and a reduction in blood glucose concentration. This suggests inhibition of glucose transport through the intestine, based also on the results observed in vitro using Caco-2 cells, where lignosulfonic acid significantly inhibited the uptake of 2-deoxyglucose. The authors suggested that lignins and lignosulfonic acid could be used in the treatment of diabetes.

**Figure 2:**
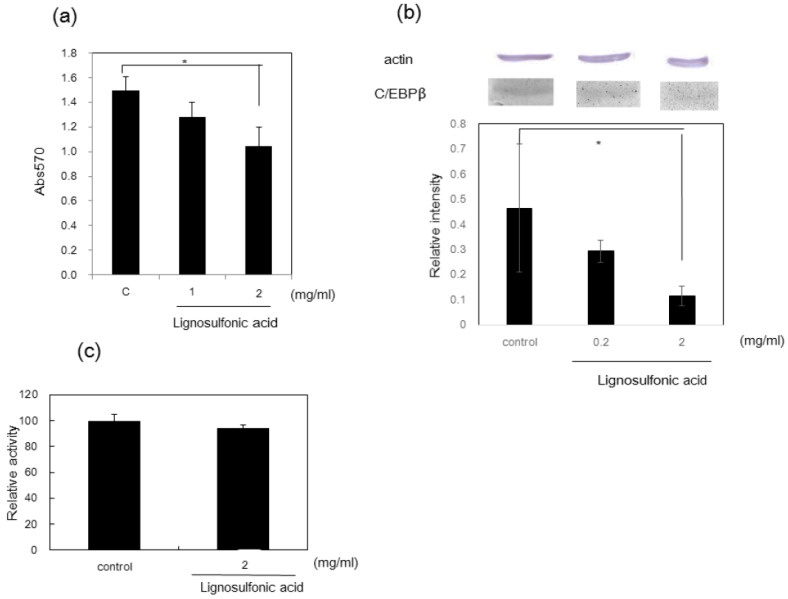
Effect of lignosulfonic acid on preadipocyte proliferation. (**a**) Lignosulfonic acid was added to 3T3-L1 preadipocyte cells at concentrations of 1.0 mg/mL and 2.0 mg/mL compared to control in the absence of lignosulfonic acid; (**b**) effect of lignosulfonic acid on the expression of C/EBP-β; (**c**) LDH content released into culture medium. Statistical significance was evaluated by Student’s *t*-test (* *p* < 0.05). (Reprinted with permission from [16]).

Other potentially beneficial effects of lignosulfonic acid have been studied for the treatment of diabetes. Lignosulfonic acid has been demonstrated to inhibit mitotic clonal expansion of 3T3-L1 preadipocyte cells in vitro (Figure 2). Moreover, feeding lignosulfonic acid to KK-Ay diabetic mice suppressed the increase of serum glucose levels seen in untreated control animals [16].

### 2.2. Lignophenols in Obesity Control

Lignophenols (LPs), which are lignin derivatives, were first isolated many years ago using a phase-separation system [17]. Their chemical structure is similar to that of natural lignin. Although LPs are known to be highly phenolic and highly stable, and also to exhibit antioxidant properties in vitro [18], their physiological role remains unclear.

**Figure 3:**
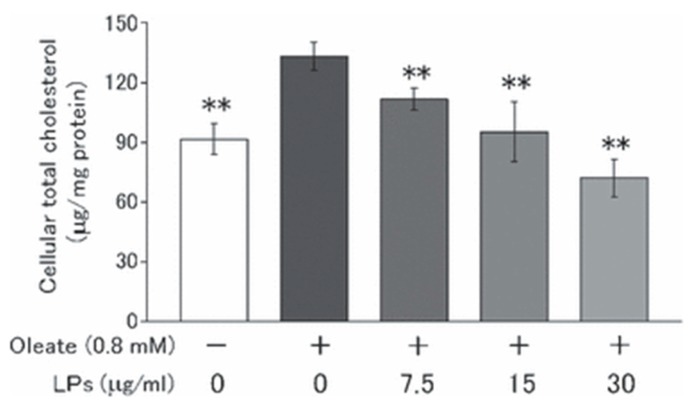
Effect of Lignophenols (LPs) on the amount of cellular total cholesterol in HepG2 cells incubated for 48 h in serum-free media containing different concentrations of LPS. The differences between means were significant at ** *p* < 0.01, compared with oleate-treated cultures (Reprinted with permission from [20]).

LPs derived from bamboo were reported to prevent hydrogen peroxide-induced cell death in vitro [19]. LPs reduced oleate-induced apolipoprotein-B secretion and sterol regulatory element binding protein-2 in HepG2 cells. Meanwhile, treatment with LPs significantly decreased oleate-induced apo-B secretion from HepG2 cells in a dose-dependent manner. At least in part, LPs may attenuate apo-B secretion by decreasing the rate of exit from the rough endoplasmic reticulum, which is controlled by the microsomal triglyceride transfer protein. Moreover, LPs reduce the amount of cholesterol in HepG2 in a dose-dependent manner (Figure 3) [20].

Moreover, LPs attenuated the excess oxidative stress, infiltration and activation of macrophages, as well as glomerular expansion in the kidneys of streptozotocin-induced diabetic rats [21]. However, there is limited information on the potential beneficial effects of a LP rich diet on diet-induced obesity. In conclusion, it has been demonstrated that LP treatment suppresses adipose tissues, plasma triglyceride levels, and hepatic expression of SREBP-1c mRNA in rats fed a high-fat diet. However, the mechanism underlying the effects of LP treatment on lipid metabolism in diet-induced obesity should be studied further, as the results may facilitate the formulation of preventive care strategies for obesity [22].

### 2.3. Lignosulfonic Acid with Antiviral Activity

Lignosulfonic acid (LA) is a low-cost lignin-derived polyanionic macromolecule. It is derived from the sulfite pulping of softwood used in the paper industry and is also used as a raw material in the production of artificial vanilla flavor, vanillin [23].

The antiviral activity of lignosulfonic acid has been studied in vivo and in vitro. Lignosulfonic acid exhibits potent and consistent broad-spectrum anti-HIV activity in the lower μM range, showing low, if any, cytotoxicity in replication and co-cultivation assays. The in vivo studies were performed in infected mice by exposing scarified skin to an HSV-2 G suspension. The development of lesions and mortality were recorded over 25 days. Primary lesions developed at the site of inoculation and the severity of the infection was scored based on the number and size of the lesions [24].

**Figure 4:**
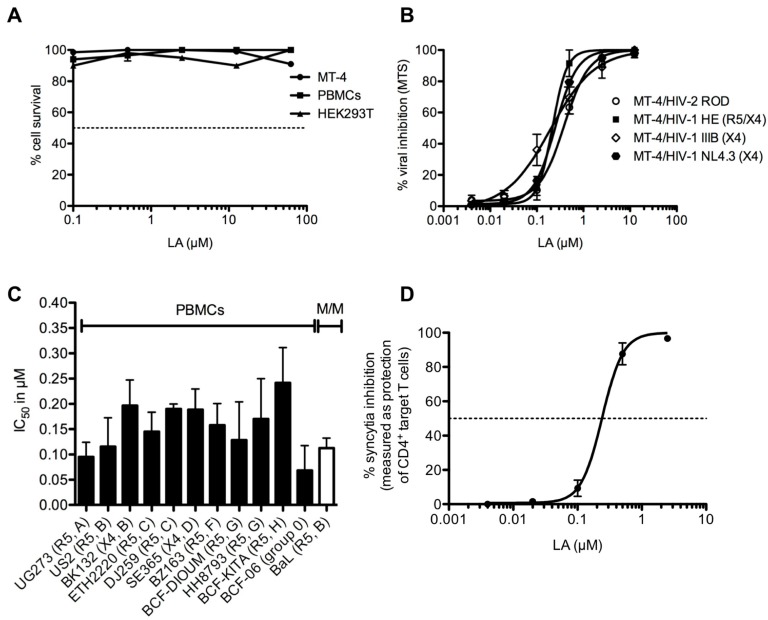
Cytotoxicity of Lignosulfonic acid (LA) on human T-cell leukemia cell line (MT-4) cells, human embryonic kidney cells (HEK293T) and peripheral blood mononuclear cells (PBMCs). (**A**) Dose dependent anti-HIV activity of LA in the CD4+ T-lymphoma cell line MT-4 against 3 laboratory HIV-1 strains (NL4.3, IIIB and HE) and 1 HIV-2 strain (ROD); (**B**) Evaluation of the IC50s of LA against various clinical isolates representing different of HIV-1 primary cells; (**C**) Dose-dependent effect of LA on the giant cell (syncytia) formation between persistently HIV-1 IIIB-infected T cells (HUT-78/IIIB) and non-infected CD4+ target SupT1 T cells; (**D**) Dashed lines correspond to 50%, (Reprinted with permission from [25]).

Different cells were also used such as a human T-cell leukemia cell line (MT-4), human embryonic kidney cells (HEK293T) and peripheral blood mononuclear cells (PBMCs) infected with HIV. The in vitro and in vivo results of inhibition of both HIV (Figure 4) and HSV transmission and infection by lignosulfonic acid, together with its high availability and safety profile, make it a very promising antiviral candidate [25].

Lignosulfonic acid also exhibits potent and broad activity against HIV-1 isolates of diverse subtypes, including two North America strains and a number of Chinese clinical isolates. Therapeutic viral reverse transcriptase inhibitor has successfully been tested in clinical trials and it has been demonstrated to be safe and effective in preventing sexual transmission of HIV-1 [26]. The synergistic effects of lignosulfonic acid in combination with a number of reverse transcriptase inhibitors, together with its low toxicity to epithelial cells, make lignosulfonic acid an interesting candidate as a topical microbicide. Topical application of lignosulfonic acid, opposed to systemic use, would avoid the low capacity to penetrate blood barriers and toxicity to lymphocytes [27]. Lignosulfonic acid shows no adverse effect on epithelial integrity and on the tight junction protein expression. Furthermore, it modulates inflammatory cytokine expression. Moreover, lignosulfonic acid did not disturb the growth of lactobacillus, an important component of vaginal microflora. Together, these results suggest that lignosulfonic acid deserves to be studied further as a topical microbicide candidate [28].

### 2.4. Lignin–Carbohydrate Complexes as Antiviral Agents and Immunomodulators

Lignin and carbohydrate molecules can be physically or chemically bonded to each other, mainly by covalent bonds. In wood, these complexes consist of ester and ether linkages through sugar hydroxyl to the α-carbanol of phenylpropane subunits in lignin. Water-soluble lignin–carbohydrate complexes (LCCs) often precipitate during digestion with polysaccharidases, and the residual sugars are more diverse than the bulk hemicellulose [29]. They can be obtained from different sources, such as sugarcane bagasse [30].

LCCs have been prepared by sequential alkaline extraction and acid precipitation from pinecones and pine nut shells, as well as from extracts of *Lentinus edodes mycelia* and *Sasa senanensis Rehder* leaves. They show excellent anti-UV activity [31,32] and so they could be used in cosmetic sun care products [33].

Pinecone LCC from *Prunella vulgaris* with a molecular weight of 8500 has been reported to show anti-herpes activity, by inhibiting viral binding and penetration [34]. More recent work suggested that LCCs from *P. anisum* show antiviral activities against the herpes simplex virus, human cytomegalovirus and the measles virus [35].

LCCs protected cells from the cytopathic effects of HIV infection and UV irradiation more efficiently than other polyphenols. Limited digestion of LCCs suggests that the lignin moiety is involved in the prominent anti-HIV activity; whereas the carbohydrate moiety may function in immunopotentiating activity through a cell surface receptor [36].

LCCs prepared from cacao husk and cacao mass also present anti-HIV activity, with that from cacao husk showing greater activity. Moreover, they synergistically enhance the superoxide anion and hydroxyl radical-scavenging activity of vitamin C, and stimulate nitric oxide generation by mouse macrophage-like cells (RAW264.7) [37]. In accordance with these findings, LCCs seem to be a chemical class that shows biological antiviral activities and immunomodulatory effects.

Two carboxylated lignins based on a 4-hydroxy cinnamic acid scaffold were synthesized using enzymatic oxidative coupling. They have been studied in HeLa cells infected with herpes simplex virus-1 (HSV-1). The two carboxylated lignins were found to inhibit HSV-1 entry into mammalian cells and to be more potent than sulfated lignins [38].

### 2.5. Low-Molecular-Weight Lignins as Anticoagulant and Anti-Emphysema Agents

An old study demonstrated the anticoagulant activity of sulfonated lignins [39]. More recently, in one approach to designing new anticoagulants with a dual hydrophobic and anionic nature, sulfated low-molecular-weight variants of lignins were prepared as functional mimetics of low-molecular-weight heparin [40].

Sulfated low-molecular-weight lignins (LMWLs) are composed of oligomeric chains of varying lengths and contain different intermonomeric linkages, such as β-*O*-4 and β-5.

Mechanistically, the sulfated LMWLs were found to act via a novel anticoagulation mechanism involving exosite II-mediated allosteric inhibition of thrombin [41].

Overall, the chemo-enzymatic origin of sulfated LMWL, coupled with dual plasmin and thrombin inhibition properties, presents novel opportunities for designing new pharmaceutical agents that could regulate complex pathologies in which both systems are known to play important roles [42].

**Figure 5:**
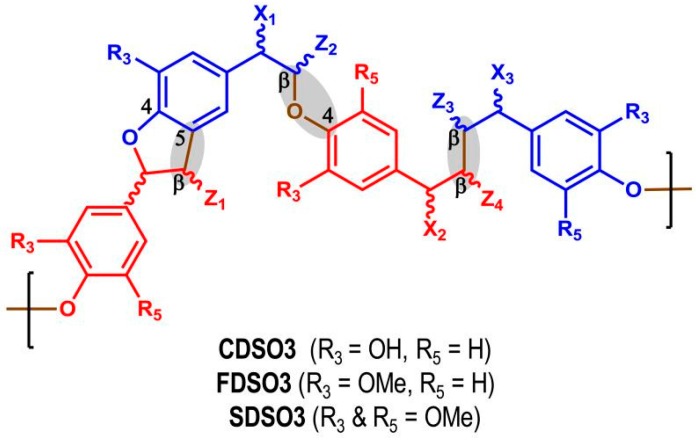
Sulfated low molecular weight lignins are complex three-dimensional oligomers obtained from enzymatic condensation of 4-hydroxycinnamic acid monomers using horseradish peroxidase followed by chemical sulfation using sulfur trioxide. The oligomers primarily contain β-*O*-4, β-5 and β–β inter-residue linkages (shown shaded). (Reprinted with permission from [43]).

Sulfated LMWLs (Figure 5) represent a totally novel class of anticoagulants, which utilize the heparin binding domain to induce allosteric inhibition of a variety of serine coagulation proteases. Although other ligands have been reported to bind to the heparin binding site of serine coagulation proteases, none has exhibited a direct anticoagulant effect [43].

Sulfated β-*O*-lignins have been recently discovered as anticoagulants. They simultaneously induce anticoagulation and antiplatelet actions by targeting exosite 2 of thrombin to reduce fibrinogen cleavage through allostery and compete with glycoprotein Iba to reduce platelet activation [44].

Based on the results obtained with LMWLs as anticoagulants with potent inhibitory effects on blood serine proteases, factor Xa and thrombin, a recent study propose the use of these lignins in the treatment of emphysema. Emphysema is one of the major pathological manifestations of chronic obstructive pulmonary disease, which causes high morbidity and mortality worldwide. Elastolysis, oxidative stress and inflammation in different lung cells have been suggested as three major pathogenic mechanisms causing emphysema [45]. Another recent study described novel unsulfated or sulfated LMWLs prepared from three 4-hydroxycinnamic acids, caffeic acid, ferulic acid and sinapic acid. They possessed potent inhibitory activity against neutrophil elastase, oxidation and inflammation in vitro [46].

### 2.6. Lignin-Based Nanoparticles for Drug Delivery

Some years ago, different authors demonstrated the capacity of nanoparticles (NPs) obtained from lignin for the controlled release of different herbicides and pesticides [47,48]. This potential can be extended to the release of different drugs in human medicine.

NPs from lignin have the advantage of being non-toxic and biodegradable, and for this reason they are suitable for drug delivery and, as stabilizers of cosmetic and pharmaceutical formulations. They can also be used in areas where they may replace more expensive and potentially toxic nanomaterials [49].

Recently, water-dispersed lignin NPs have been developed which can be used to stabilize Pickering emulsions as well as to carry silver ions in antimicrobial applications. These could also be used in drug delivery applications that may include cancer treatments [50].

Lignin is one of the most abundant biopolymers in nature; it is also naturally biodegradable, biocompatible and presents very good stability. Together, all this makes lignin an ideal precursor for the development of environmentally friendly nanomaterials, in contrast to many other NPs used in anticancer therapy [51].

**Figure 6:**
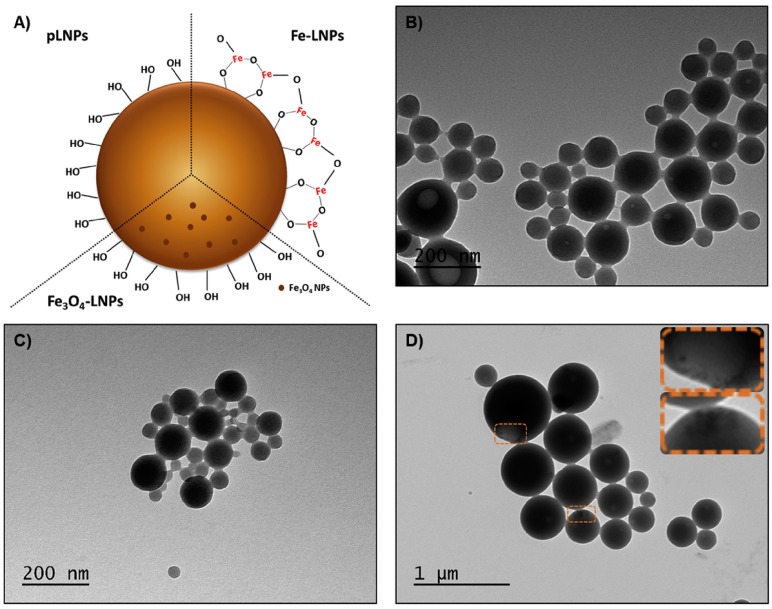
Schematic representation of the three types of lignin nanoparticles (LNPs) (**A**), and TEM images of (**B**) pLNPs, (**C**) Fe-LNPs and (**D**) Fe_3_O_4_-LNPs and magnification of the Fe_3_O_4_ NPs inside the LNPs. (Reprinted with permission from [52]).

In addition to their biocompatibility and very good stability, innovative lignin NPs exhibit other important features for drug delivery and biomedical applications (Figure 6). These include the capacity to carry hydrophobic drugs and sustain their release, and good cellular interactions. As a result of their surface structure, lignin NPs can be modified with targeting moieties in order to increase cellular interaction with specific cells, e.g., for cancer therapy. Also, pH-sensitive polymers can be added to lignin-based NPs to allow pH-responsive drug release and possibly loading of hydrophilic drugs. NPs loaded with drugs that are only poorly water soluble, such as benzazulene and sorafenib, or with a water-soluble anticancer drug such as capecitabine, showed inhibitory effects on different cancer cells including MDA-MB-231 and MCF-7 (human breast cancer cell lines), PC3-MM2 (a prostate cancer cell line), Caco-2 (colorectal adenocarcinoma). EA.hy926, a non-tumor cell line, was used for comparative purposes [52].

Another demonstrated application of lignin NPs is sun protection. Lignins were obtained from *A. tequilana Weber* bagasse by soda and organosolv pulping, and then two lignin NPs were synthesized.

**Figure 7:**
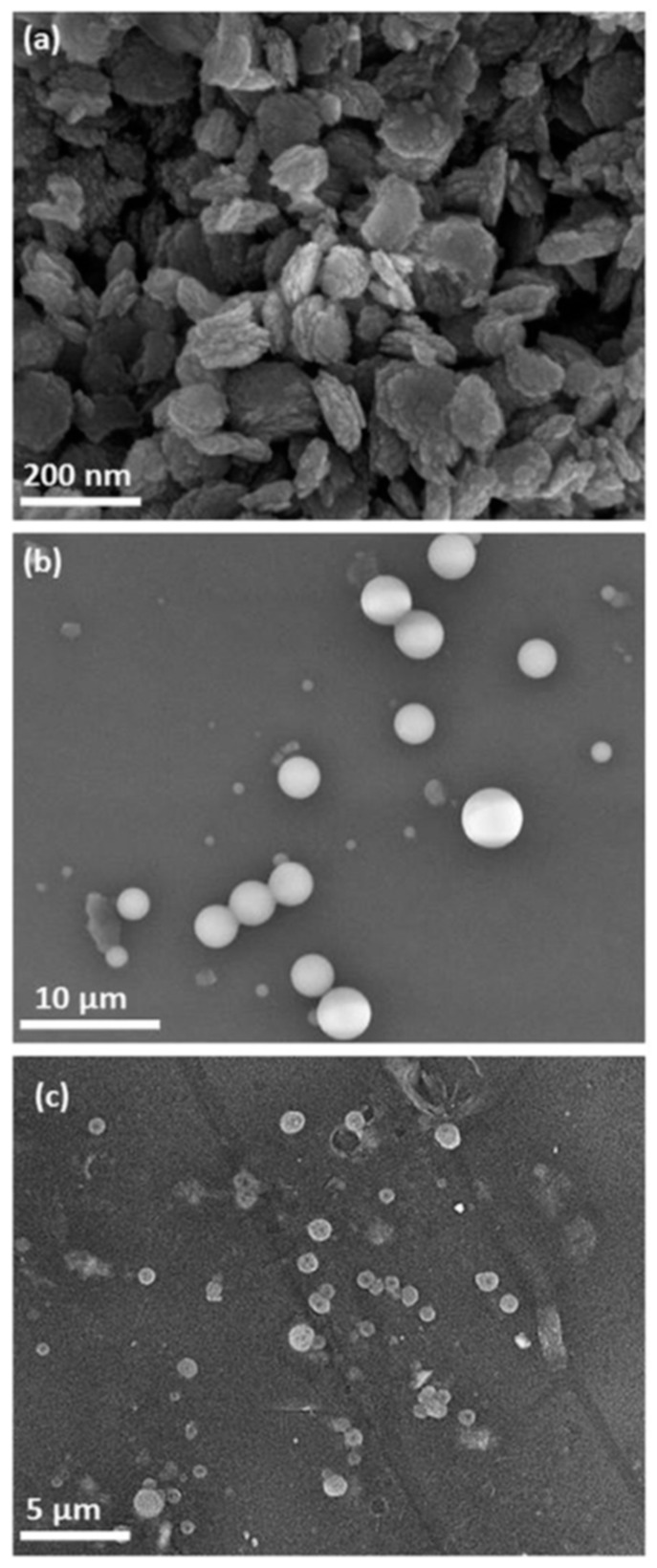
Scanning electron microcopy (SEM) images of (**a**) Zinc oxide nanoparticles (**b**) ligninnanoparticles organosolv and (**c**) lignin nanoparticles soda. (Reprinted with permission from [53].

The UV absorption of organosolv-type NPs in combination with a neutral vehicle shows similar levels of protection (as expressed by the sun protection factor: SPF) to ZnO NPs; and they were higher than those for soda lignin NPs. The addition of ZnO NPs together with lignin resulted in an additive enhancement of SPF in these formulations, especially with lignin from soda pulping (Figure 7).

Bulk lignin absorbs only one-fourth of the UV intensity absorbed by lignin NPs. This demonstrates the effect of the smaller size on the UV absorption properties of lignin. Although nanosized lignin formulation still requires optimization, initial results suggest a viable new option from sustainable resources for use in photoprotection [53].

Lignin can be coated on silverNPs in order to increase their antibacterial activity. For this application, silica/lignin hybrid materials were first obtained. The silica surface, suitably modified using *N*-(2-aminoethyl)-3-aminopropyltrimethoxysilane, and kraft lignin, activated with a solution of a strong oxidizing agent, were bound together in a chemically permanent fashion. Next, silver NPs were grafted onto the surface of the silica/lignin hybrids. Presumably the chemical or ionic nature of the hybrid-silver nanoparticles (NP) bonds provides the high stability of silver NPs, as well as extended longevity of the antibacterial activity. The use of a relatively cheap inorganic material, in combination with lignin, which is a waste product of the paper industry, thus made it possible to create a hybrid material which is as biocompatible as pure silica, but even more cost-efficient. With its surface effectively functionalized with silver NPs, the hybrid material demonstrated antibacterial activity against all the tested species of bacteria. The strongest antimicrobial effect was observed in the case of *P. aeruginosa*, an opportunistic human pathogen [54].

Chitosan-based delivery systems have been used for the improved delivery and controlled release of peptides, proteins, oligonucleotides and plasmids, by protecting the macromolecules from enzymatic degradation [55]. For the production of chitosan NPs, the addition of an anionic compound is the most common technique, but this is limited by the ionic interaction between chitosan and the anionic compound. For this reason, the use of sulfonated lignin was proposed as a counter ion polymer, to increase the stability of chitosan NPs [56] and to provide antimicrobial activity [57].

The beneficial effects of lignosulfonate complexes on chitosan NPs were characterized, showing greater stability to lysozyme degradation, biocompatibility with human cells and antimicrobial activity against both Gram-negative and Gram-positive bacteria. The recovery of fibroblasts from cytotoxic effects has been demonstrated, indicating that these NPs can safely be applied to human skin [56].

### 2.7. Other Lignins in Drug Delivery

Lignin from sugarcane has been used to deliver methrotrexate, a drug used in the treatment of rheumatoid arthritis in a rat model. Lignin was deemed to disperse into the intercapillary blood vessels, transporting the drug towards hard-to-target inflamed tissues. In the methrotrexate–lignin drug carrier, the interlayer sheet structure of the lignin matrix, together with the dispensed angstrom-sized cellulosic crystals over the lignin, result in methotrexate being released in cells. Moreover, the methrotrexate-lignin drug delivery system repaired seminiferous tubules [58].

A xanthan and lignin epoxy-modified resin (LER) mixture was crosslinked using epichlorohydrin as the crosslinking agent, leading to superabsorbent hydrogels with a fast swelling rate in aqueous mediums. These hydrogels were tested as carries by the loading/delivery behavior of bisoprolol fumarate, a drug that belongs to the group of medicines called beta-blockers that are used in the treatment of stable chronic heart failure. The amount of drug loaded into the polymer networks was found to range between 14.4% and 19.2%. Drug release was retarded and the release mechanism of the active principle was found to depend on the composition of the matrix [59].

An interesting review of the research performed on the lignin-based controlled release of bioactive materials concluded that the transformed lignin materials may be helpful for studies of water-insoluble drug loading and their release. This can be considered further potential for future value-adding applications of lignins in biomedical fields [60].

### 2.8. Lignin Metabolites in Cancer

The chaga mushroom, Inonotus obliquus, has been recognized as a remedy for cancer, gastritis, ulcers, and tuberculosis of the bones since the 16th century [61]. Two homogenous carbohydrate–lignin metabolites from water-soluble fractions were obtained and their antiproliferative activity was studied.

Chemical and spectral characterizations indicated that both were lignin metabolites in which carbohydrate constituents made hydrophobic lignin highly water-soluble. Cytotoxicity tests showed that these lignin derivatives induced cell apoptosis, which was largely cell-cycle independent. In addition, both carbohydrate-lignin metabolites inhibited the activation of the nuclear transcription factor NF-κB in cancer cells [62].

## 3. Conclusions

Lignins are obtained from various plants or biomass via a range of processes. However, they are generally obtained as a by-product from the black liquor of chemical pulping processes. Lignins are currently used to produce energy, through great efforts are being made to find new applications of them, since they are relatively cheap. Since these readily available plant-derived materials are biocompatible, research on their potential use in biomedical applications has increased in recent years. In many cases the application of lignins is based on their antioxidant capacity. The applications of lignins studied include their antiviral and antitumor effects. Moreover, the use of lignins in the development of NPs for drug delivery is increasing. Table 1 shows the main pharmacological activities of lignins and their derivatives.

## Figures and Tables

**Table 1:** Main pharmacological activities of lignins and their derivatives.

Compound	Effect	Mechanism	Experimental Model	Reference
Alkali lignin	Antidiabetic	α-amylase inhibition In vitro decreased glucose diffusion	In vitro glucose movement	[14]
Lignosulfonic acid	Antidiabetic	Inhibitor of α-glucosidase Decrease blood glycemia	In vitro inhibitor of α-glucosidase Rat in vivo	[15]
Lignophenols	Obesity control	Decrease oleate-induced apo-B secretion	HepG2 in vitro	[20]
Lignophenols	Obesity control	Decrease plasma triglyceride levels	Rats fed a high-fat diet	[21]
Lignosulfonic acid	Antiviral activity	Inhibition of the replication of herpes simplex virus (HSV)	Infected mice by exposing scarified skin to an HSV-2 G	[24]
Lignosulfonic acid (LA)	Antiviral activity	LA mainly binds to the HIV-1 envelope glycoproteins	In vitro cells: T-lymphoma cell lines, HEK293T, HUT-78, Monocyte-derived dendritic cells	[25]
Lignin–carbohydrate complexes	Antiviral activity	Inhibiting viral binding and penetration	Vero cells infected with herpes simplex virus	[35]
Lignin–carbohydrate complexes	Antiviral activity	Inhibiting viral binding and penetration	HeLa cells infected with herpes simplex virus	[38]
Sulfated low-molecular-weight lignins	Anticoagulant	Inhibition of thrombin	Binding to thrombin	[43]
Sulfated low-molecular-weight lignins	Anticoagulant	Allosteric inhibition of thrombin	Whole blood thromboelastography, hemostasis analysis and mouse arterial thrombosis models	[44]
Sulfated low-molecular-weight lignins	Antiemphysema	Elastase, oxidation and inflammation inhibition	In vitro human alveolar A549 and bronchial Calu-3 epithelial cells	[46]

## Supplementary Materials

Supplementary materials can be found at www.mdpi.com/1422-0067/18/6/1219/s1.
