# Supplementary material for: Lignins and Their Derivatives with Beneficial Effects on Human Health
Source: Int J Mol Sci. 2017 Jun 7;18(6):1219. doi: 10.3390/ijms18061219 (PMC5486042; doi:10.3390/ijms18061219)
Supplement: Supplementary file 1 [file ijms-18-01219-s001.zip › permisos/Figure 1. Zakseski.pdf]

Copyright Clearance Center RightsLink®

Home | Account Info | Help

ACS Publications
Most Trusted. Most Cited. Most Read.

Title: The Catalytic Valorization of Lignin for the Production of Renewable Chemicals

Author: Joseph Zakzeski, Pieter C. A. Bruijnincx, Anna L. Jongerius, et al

Publication: Chemical Reviews

Publisher: American Chemical Society

Date: Jun 1, 2010

Copyright © 2010, American Chemical Society

Logged in as:

Pilar Vinardell

Account #:
3000631795

LOGOUT

PERMISSION/LICENSE IS GRANTED FOR YOUR ORDER AT NO CHARGE

This type of permission/license, instead of the standard Terms & Conditions, is sent to you because no fee is being charged for your order. Please note the following:

- Permission is granted for your request in both print and electronic formats, and translations.
- If figures and/or tables were requested, they may be adapted or used in part.
- Please print this page for your records and send a copy of it to your publisher/graduate school.
- Appropriate credit for the requested material should be given as follows: "Reprinted (adapted) with permission from (COMPLETE REFERENCE CITATION). Copyright (YEAR) American Chemical Society." Insert appropriate information in place of the capitalized words.
- One-time permission is granted only for the use specified in your request. No additional uses are granted (such as derivative works or other editions). For any other uses, please submit a new request.

If credit is given to another source for the material you requested, permission must be obtained from that source.

BACK | CLOSE WINDOW

Copyright © 2017 Copyright Clearance Center, Inc. All Rights Reserved. Privacy statement. Terms and Conditions.
Comments? We would like to hear from you. E-mail us at customercare@copyright.com
