# Supplementary material for: Lignins and Their Derivatives with Beneficial Effects on Human Health
Source: Int J Mol Sci. 2017 Jun 7;18(6):1219. doi: 10.3390/ijms18061219 (PMC5486042; doi:10.3390/ijms18061219)
Supplement: Supplementary file 1 [file ijms-18-01219-s001.zip › permisos/Figure 3 Norikura 2010.pdf]

JOHN WILEY AND SONS LICENSE
TERMS AND CONDITIONS

Mar 03, 2017

This Agreement between Pilar Vinardell ("You") and John Wiley and Sons ("John Wiley and Sons") consists of your license details and the terms and conditions provided by John Wiley and Sons and Copyright Clearance Center.

License Number	4061280689165
License date	Mar 03, 2017
Licensed Content Publisher	John Wiley and Sons
Licensed Content Publication	Basic & Clinical Pharmacology & Toxicology
Licensed Content Title	Lignophenols Decrease Oleate-Induced Apolipoprotein-B Secretion in HepG2 Cells
Licensed Content Author	Toshio Norikura,Yuuka Mukai,Shuzo Fujita,Keigo Mikame,Masamitsu Funaoka,Shin Sato
Licensed Content Date	Apr 20, 2010
Licensed Content Pages	5
Type of use	Journal/Magazine
Requestor type	University/Academic
Is the reuse sponsored by or associated with a pharmaceutical or medical products company?	no
Format	Print
Portion	Figure/table
Number of figures/tables	1
Original Wiley figure/table number(s)	Figure 5
Will you be translating?	No
Circulation	1000
Title of new article	Lignins and their derivatives with beneficial effects on human health
Publication the new article is in	nternational Journal of Molecular Sciences
Publisher of new article	MDPI AG
Author of new article	Vinardell MP, Mitjans M
Expected publication date of new article	Oct 2017
Estimated size of new article (pages)	8
Requestor Location	Pilar Vinardell<br>Facultat de Farmacia<br>Av Joan XXIII s/n<br><br>Barcelona, 08028<br>Spain<br>Attn: Pilar Vinardell
