# Supplementary material for: Lignins and Their Derivatives with Beneficial Effects on Human Health
Source: Int J Mol Sci. 2017 Jun 7;18(6):1219. doi: 10.3390/ijms18061219 (PMC5486042; doi:10.3390/ijms18061219)
Supplement: Supplementary file 1 [file ijms-18-01219-s001.zip › permisos/Figure 4 Gordts 2015.pdf]

# The Low-Cost Compound Lignosulfonic Acid (LA) Exhibits Broad-Spectrum Anti-HIV and Anti-HSV Activity and Has Potential for Microbicidal Applications

Stephanie C. Gordts,[#1] Geoffrey Férir,[#1,*] Thomas D'huys,[1] Mariya I. Petrova,[2,3] Sarah Lebeer,[2,3] Robert Snoeck,[1] Graciela Andrei,[1] and Dominique Schols[1]
Roberto F. Speck, Editor
Author information ► Article notes ► Copyright and License information ▼
Copyright notice
This is an open access article distributed under the terms of the Creative Commons Attribution License, which permits unrestricted use, distribution, and reproduction in any medium, provided the original author and source are credited
