# Supplementary material for: Lignins and Their Derivatives with Beneficial Effects on Human Health
Source: Int J Mol Sci. 2017 Jun 7;18(6):1219. doi: 10.3390/ijms18061219 (PMC5486042; doi:10.3390/ijms18061219)
Supplement: Supplementary file 1 [file ijms-18-01219-s001.zip › permisos/Figure 6 Figuereido 2017.pdf]

ELSEVIER LICENSE
TERMS AND CONDITIONS

Mar 02, 2017

This Agreement between Pilar Vinardell ("You") and Elsevier ("Elsevier") consists of your license details and the terms and conditions provided by Elsevier and Copyright Clearance Center.

License Number	4060790101381
License date	Mar 02, 2017
Licensed Content Publisher	Elsevier
Licensed Content Publication	Biomaterials
Licensed Content Title	In vitro evaluation of biodegradable lignin-based nanoparticles for drug delivery and enhanced antiproliferation effect in cancer cells
Licensed Content Author	Patrícia Figueiredo,Kalle Lintinen,Alexandros Kiriazis,Ville Hynninen,Zehua Liu,Tomás Bauleth-Ramos,Antti Rahikkala,Alexandra Correia,Tomáš Kohout,Bruno Sarmento,Jari Yli-Kauhaluoma,Jouni Hirvonen,Olli Ikkala,Mauri A. Kostiainen,Hélder A. Santos
Licensed Content Date	March 2017
Licensed Content Volume	121
Licensed Content Issue	n/a
Licensed Content Pages	12
Start Page	97
End Page	108
Type of Use	reuse in a journal/magazine
Requestor type	author of new work
Intended publisher of new work	MDPI AG
Portion	figures/tables/illustrations
Number of figures/tables /illustrations	1
Format	print
Are you the author of this Elsevier article?	No
Will you be translating?	No
Order reference number	
Original figure numbers	Figure 1
Title of the article	Lignins and their derivatives with beneficial effects on human health
Publication new article is in	nternational Journal of Molecular Sciences
Publisher of the new article	MDPI AG
Author of new article	Vinardell MP, Mitjans M
Expected publication date	Oct 2017
