# Supplementary material for: Lignins and Their Derivatives with Beneficial Effects on Human Health
Source: Int J Mol Sci. 2017 Jun 7;18(6):1219. doi: 10.3390/ijms18061219 (PMC5486042; doi:10.3390/ijms18061219)
Supplement: Supplementary file 1 [file ijms-18-01219-s001.zip › permisos/Figure 7 Guitierrez.pdf]

ELSEVIER LICENSE
TERMS AND CONDITIONS

Mar 02, 2017

This Agreement between Pilar Vinardell ("You") and Elsevier ("Elsevier") consists of your license details and the terms and conditions provided by Elsevier and Copyright Clearance Center.

License Number	4060790630922
License date	Mar 02, 2017
Licensed Content Publisher	Elsevier
Licensed Content Publication	Journal of Photochemistry and Photobiology B: Biology
Licensed Content Title	Use of Agave tequilana-lignin and zinc oxide nanoparticles for skin photoprotection
Licensed Content Author	José Manuel Gutiérrez-Hernández,Alfredo Escalante,Raquel Nalleli Murillo-Vázquez,Ezequiel Delgado,Francisco Javier González,Guillermo Toríz
Licensed Content Date	October 2016
Licensed Content Volume	163
Licensed Content Issue	n/a
Licensed Content Pages	6
Start Page	156
End Page	161
Type of Use	reuse in a journal/magazine
Requestor type	author of new work
Intended publisher of new work	MDPI AG
Portion	figures/tables/illustrations
Number of figures/tables /illustrations	1
Format	print
Are you the author of this Elsevier article?	No
Will you be translating?	No
Order reference number	
Original figure numbers	Figure 1
Title of the article	Lignins and their derivatives with beneficial effects on human health
Publication new article is in	nternational Journal of Molecular Sciences
Publisher of the new article	MDPI AG
Author of new article	Vinardell MP, Mitjans M
Expected publication date	Oct 2017
Estimated size of new article (number of pages)	8
